# 396. Disparities in SARS-CoV-2 Antibody Prevalence: Findings from a Citywide Serosurvey in Holyoke, Massachusetts, November 2020–January 2021

**DOI:** 10.1093/ofid/ofab466.597

**Published:** 2021-12-04

**Authors:** Wilfredo Matias, Isabel Fulcher, Cody Nolan, Yodeline Guillaume, Jack Zhu, Francisco Molano, Elizabeth Uceta, Shannon Collins, Damien Slater, Vanessa Sanchez, Serina Moheed, Jason Harris, Richelle Charles, Ryan Paxton, Sean Gonsalves, Molly Franke, Louise Ivers

**Affiliations:** 1 Mass GeneralBrigham, Boston, Massachusetts; 2 Harvard Data Science Initiative, Boston, Massachusetts; 3 Brigham and Women’s Hospital, Boston, Massachusetts; 4 Massachusetts General Hospital, Boston, Massachusetts; 5 smoheed@mgh.harvard.edu, Boston, Massachusetts; 6 Holyoke Board of Health, Holyoke, Massachusetts; 7 Harvard Medical School, Boston, Massachusetts

## Abstract

**Background:**

Seroprevalence studies are important tools to estimate the prevalence of prior or recent SARS-CoV-2 infections. This information is critical for identifying hotspots and high-risk groups and informing public health responses to the COVID-19 pandemic. We conducted a city-level seroprevalence study in Holyoke, Massachusetts to estimate the seroprevalence of SARS-CoV-2 antibodies and risk factors for seropositivity.

**Methods:**

We invited inhabitants of 2,000 randomly sampled addresses to participate between November 5 and December 31, 2020. Participants completed questionnaires measuring sociodemographic and health characteristics, and COVID-19 exposure history, and provided dried blood spots for measurement of SARS-CoV-2 IgG and IgM antibodies. To calculate total and group seroprevalence estimates, inverse probability of response weights were constructed based on age, gender, race/ethnicity and census tract to ensure estimates represented the city’s population.

**Results:**

We enrolled 280 households including 472 individuals. 328 underwent antibody testing. The citywide weighted seroprevalence of SARS-CoV-2 IgG or IgM was 13.9% (95%CI 7.8 - 21.8) compared to 9.8% based on publicly reported case counts. Seroprevalence was 16.8% (95%CI 5.7 – 28.0) among individuals identifying as Hispanic compared to 8.9% (95%CI 3.0 - 14.7) among those identifying as White. Seroprevalence was 20.7% (95%CI 2.2 – 39.2) for ages 0-19; 13.8% (95%CI 5.6 – 22) for ages 20 – 44; 9.6% (95%CI 0 – 20.5) for ages 45 – 59; 4.8% (95%CI 0 – 10.2) for ages 60 – 84; and 42.9% (95%CI 0 – 100) for ages >85.

Table 1. Seroprevalence by antibody positivity profile

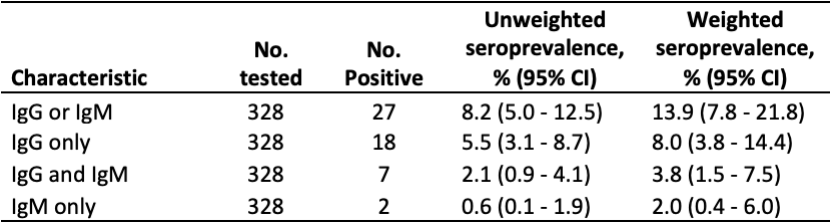

Table 2. Unweighted and weighted seroprevalence by sociodemographic characteristics

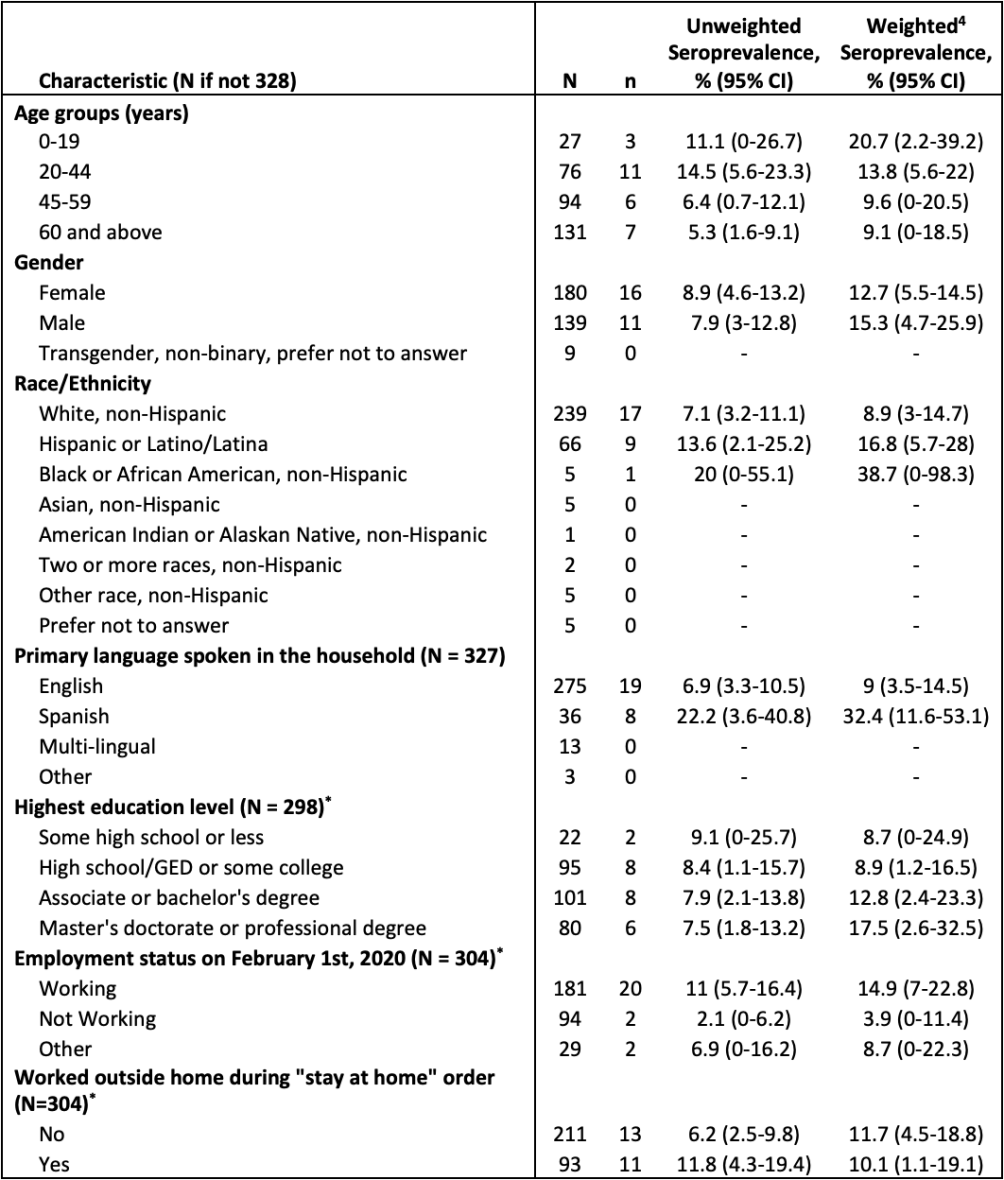

Figure 1. Seroprevalence by Medical, Symptom, Testing and Exposure History.

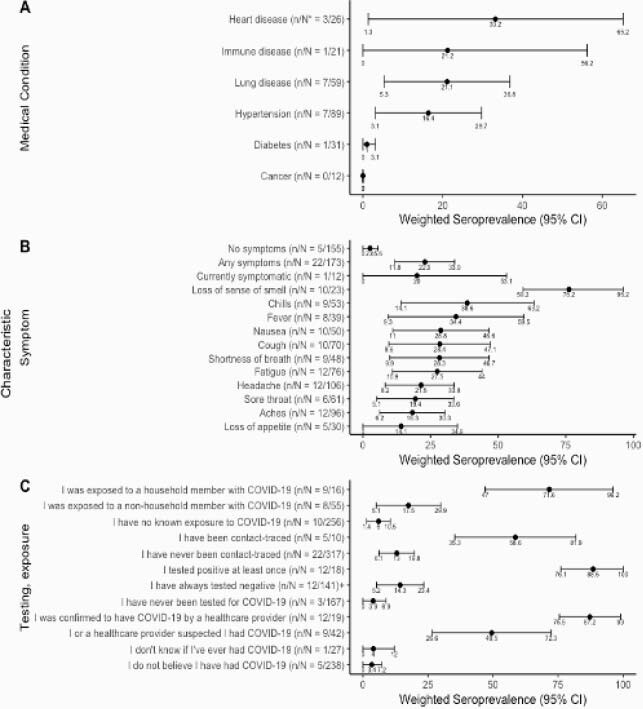

**Conclusion:**

The measured SARS-CoV-2 seroprevalence in Holyoke was only 13.9% during the second surge of SARS-CoV-2 in this region, far from accepted thresholds for “herd immunity” and highlighting the need for expanding vaccination. Individuals identifying as Hispanic were at high risk of prior infection. Subsequent community-level serosurveys are necessary to guide local responses to the SARS-CoV-2 pandemic.

**Disclosures:**

**All Authors**: No reported disclosures

